# Ten Year Follow-Up After Explantation of the Duodenal-jejunal Bypass Liner

**DOI:** 10.1007/s11695-025-08291-9

**Published:** 2025-09-26

**Authors:** Fenna M. M. Beeren, Marcel J. M. Groenen, Eric J. Hazebroek, Peter D. Siersema

**Affiliations:** 1 Rijnstate Hospital, Arnhem, Netherlands; 2 Radboud University Nijmegen Medical Centre, Nijmegen, Netherlands; 3 Erasmus MC, Rotterdam, Netherlands

**Keywords:** Duodenal-jejunal bypass liner, Long-term effects, Obesity, Diabetes mellitus type 2

## Abstract

**Background:**

The duodenal-jejunal bypass liner (DJBL) is a treatment for weight loss and diabetes management. While its short-term benefits are known, long-term outcomes of this endoscopic procedure remain largely unknown. This study investigates the long-term effects of DJBL placement on weight, diabetes, and quality of life (QoL) over a period of approximately 10 years post-explantation.

**Methods:**

A cross-sectional follow-up study was conducted in 103 former DJBL patients who had DJBL implantation (also known as the Endobarrier) between 2011 and 2014. Participants completed a questionnaire assessing their health, weight, lifestyle, diabetes control, and QoL. Data were compared to original cohort results.

**Results:**

After approximately 10 years, weight, BMI, and HbA1c levels remained significantly improved compared to the time of DJBL explantation (p < 0.05). Of the respondents, 33 (32%) had undergone metabolic bariatric surgery (MBS) post-explantation, with significant reductions in weight and BMI observed in this group. MBS was associated with better diabetes control and higher QoL scores compared to non-MBS patients. No significant differences in diabetes-related complications were seen between the MBS and non-MBS groups. GLP-1 agonists use was associated with a higher BMI but did not significantly affect weight, diabetes control, or QoL outcomes.

**Conclusions:**

Although DJBL treatment has some sustained benefits regarding weight and diabetes management, these effects are limited without further weight-reducing interventions. MBS following DJBL explantation leads to more substantial weight loss and improved diabetes outcome, highlighting its complementary role after DJBL treatment.

## Introduction

Overweight and obesity are increasingly prevalent worldwide, contributing to lifestyle-related diseases, such as cardiovascular diseases and type 2 diabetes (T2D). Consequently, various therapeutic methods to reduce weight are continuously being investigated. Common approaches include lifestyle interventions, pharmacotherapy, and metabolic bariatric surgery (MBS). A less commonly used technique is the duodenal-jejunal bypass liner (DJBL), also known as the Endobarrier (EndoBarrier®, GI Dynamics, Lexington, Massachusetts, USA).

**Fig. 1:**
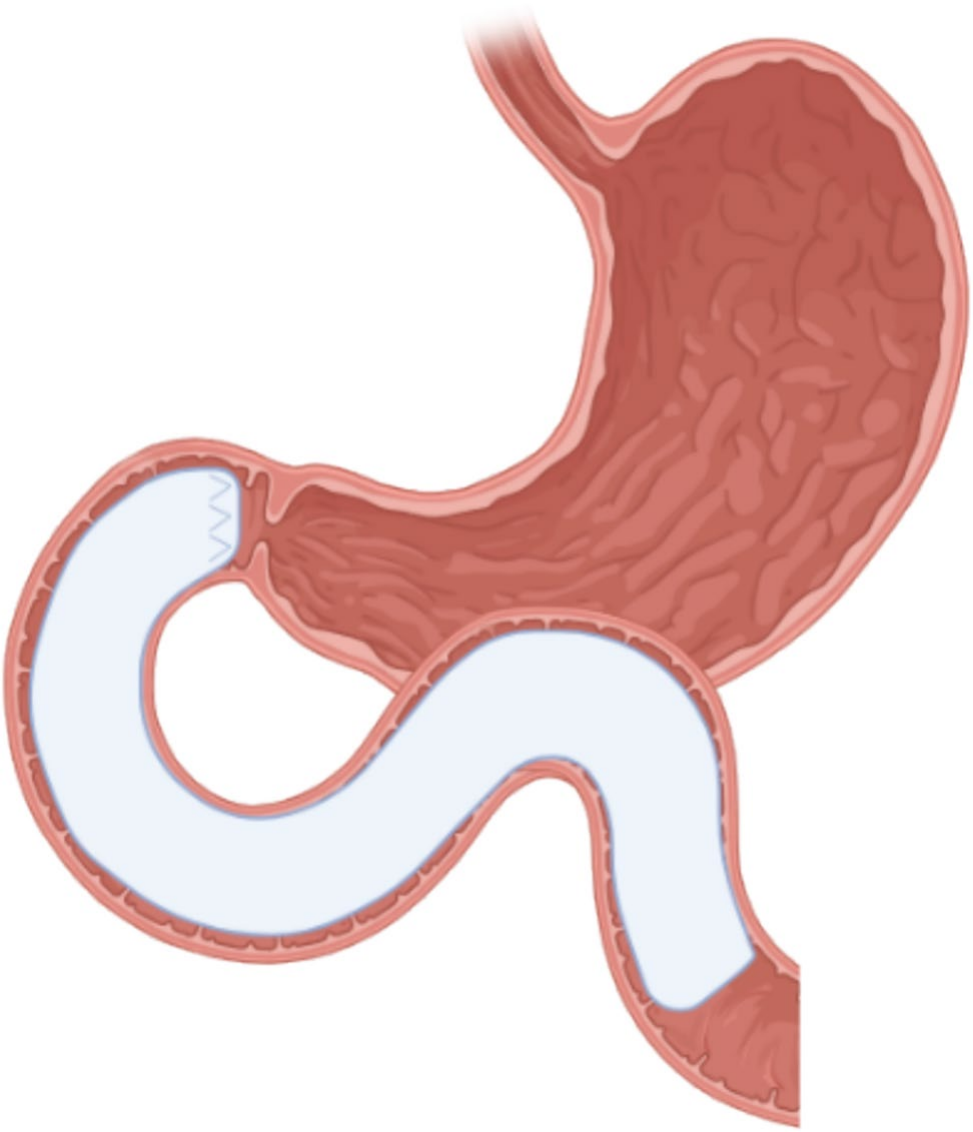
Schematic representation of the Duodenal-Jejunal Bypass Liner in situ

The DJBL is a 60 cm long, single-use, flexible fluoropolymer sleeve that is endoscopically implanted in the duodenum (Fig. 1). The device is delivered via an over-the-wire system and anchored in the duodenal bulb, proximal to the papilla of Vater, using small barbed anchors. The device is designed to remain in situ for a maximum period of 12 months. Removal is performed endoscopically by collapsing the anchor and retracting the sleeve. The DJBL functions by allowing ingested nutrients to pass through the sleeve from the stomach directly into the jejunum, thereby bypassing the duodenum. Simultaneously, bile and pancreatic secretions flow externally along the sleeve. As a result, nutrients do not come into contact with the intestinal mucosa or digestive secretions until they reach the jejunum, mimicking the duodenal-jejunal exclusion portion of a Roux-en-Y gastric bypass (RYGB), contributing to both weight loss and metabolic improvements [1–3].

Previous studies have shown DJBL’s short- to medium-term (3–4 years) benefits on weight loss and T2D management. The First National Health Service (NHS) Endobarrier Service reported that, after three years, 33/45 participants (73%) had sustained weight and HbA1c improvements [4]. Similarly, Van Rijn et al. followed 15 patients for a period of four years, with a total weight loss of 9.1% (95% CI 4.0–13.3%) after 12 months and 2.2% (95% CI −1.3–5.6%) after four years. However, 4 of 15 patients underwent RYGB during the four-year follow-up, and 1 patient was treated with a laparoscopic band, suggesting that for some individuals DJBL treatment may serve as a bridge rather than an alternative to MBS [5].

Despite its potential benefits, safety concerns such as migration, gastrointestinal bleeding, and hepatic abscesses, led to the suspension of clinical trials [6]. Nevertheless, new versions of the DJBL are being developed, with the same working mechanism while reducing adverse events. Therefore, it is important to investigate whether patients who previously had a DJBL, eventually underwent MBS at some stage. This will help gaining insight in positioning this endoscopic device as a temporary treatment for weight reduction or a preventive method for undergoing a more invasive weight-reducing intervention. In addition, it allows comparison of associated medical problems between patients who ultimately underwent MBS and those who did not.

This study aims to assess long-term outcomes of patients following 10 years after DJBL explantation.

## Materials and Methods

This is a cross-sectional follow-up study of a prospective observational cohort, in which patients had a DJBL implanted [1]. All patients who were treated with the DJBL at Rijnstate hospital, Arnhem, The Netherlands between 2011 and 2014 were included. Most of them (97.5%) participated in the original cohort study. These were adult patients with a BMI of 28–45 kg/m^2^ and T2D.

202 former DJBL patients received a questionnaire, which included questions regarding their previous experiences with the DJBL, current health and lifestyle status, any metabolic bariatric procedure they might have undergone at a later stage, and quality of life (QoL). Several attempts were done to reach non-responding patients. All procedures performed in studies involving human participants were in accordance with the ethical standards of the institutional and/or national research committee and with the 1964 Helsinki declaration and its later amendments or comparable ethical standards.

QoL was measured using the EuroQol-5Dimensions-5Levels (EQ-5D-5L) questionnaire, which is a standardized instrument for measuring generic health-related quality of life. It encompasses 5 dimensions—mobility, self-care, usual activities, pain/discomfort and anxiety/depression—each classified into 5 levels of severity, ranging from no problems to extreme problems/inability to perform [7]. The questionnaire also includes a Visual Analogue Scale (EQ VAS), which allows respondents to rate their current health on a scale from 0 (worst imaginable health) to 100 (best imaginable health). In this study, the EQ VAS was used as a summary measure of self-rated health. The five individual domains were analyzed separately based on reported severity levels.

### Statistics

Survey results were compared to the original cohort using IBM SPSS Statistics for Windows, version 29.1 (Armonk, NY, IBM Corp.). Data were presented as means with standard deviation, medians with range, or n with percentages, as appropriate. Normality was tested using the Shapiro–Wilk test and Kolmogorov–Smirnov test. Statistical significance was set at P < 0.05. Differences between the original cohort and the survey group were analyzed using a one sample t-test. Comparisons between subgroups were analyzed using a one sample T-test, independent T-test or Chi square test, if normally distributed, and a Mann–Whitney U test or Wilcoxon Signed Ranks test, if not normally distributed.

No correction for multiple comparisons was applied, as subgroup analyses were based on predefined hypotheses informed by clinical expectations. Applying a correction such as Bonferroni was therefore considered unnecessarily conservative and likely to increase the risk of type II errors.

## Results

The response rate to the questionnaire was 51%, mainly due to outdated contact information (3%) and illness or death (1.5%). Baseline characteristics are shown in Table 1. Mean age at the time of the questionnaire was 63 (± 7) years and mean weight was 89.9 (± 16.9) kg. All patients had T2D (mean duration of 22 years), and 55 patients (54%) had no T2D-related complications.

**Table 1:** Characteristics compared to Betzel et al

	Survey group (*n* = 103)	Betzel et al. baseline (n = 185)	Betzel et al. 12 months (at DJBL explantation) (*n* = 185)	*p* value
Age (years)	63 ± 7	52 ± 8	53 ± 8	
Female	50 (50%)	90 (48.6%)	90 (48.6%)	
Body weight (kg)	89.9 ± 16.9	107 ± 18	94.3 ± 16.7	p = 0.015
BMI (kg/m^2^)	29.6 ± 4.0	35.1 ± 4.3	30.9 ± 4.3	p = 0.004
Duration T2D (years)	22 (3–44)	8 (1–36)	9 (1–36)	
HbA1c (mmol/mol)	60 ± 12.8	67 ± 16	61	p = 0.043
Medication use				
Metformin	52 (50.5%)	156 (84%)	154 (83%)	
Sulfonylurea	28 (27.2%)	115 (63%)	98 (53%)	
DPP4-inhibitor	2 (1,9%)			
GLP-1 agonist	35 (34.0%)	21 (11.5%)	24 (13%)	
SGLT2 inhibitor	14 (13.6%)			
Insulin	40 (38.8%)	70 (38%)	46 (25%)	

Values are means with standard deviation, medians with range in parentheses, or n with percents in parentheses

*T2D* Type 2 Diabetes, DPP4-inhibitor: Dipeptidyl Petidase-3 inhibitor, GLP-1 agonist: Glucacon Like Peptide-1 agonist, SGLT2 inhibtor: Sodium-Glucose Linked Transporter 2 inhibitor

**Fig. 2:**
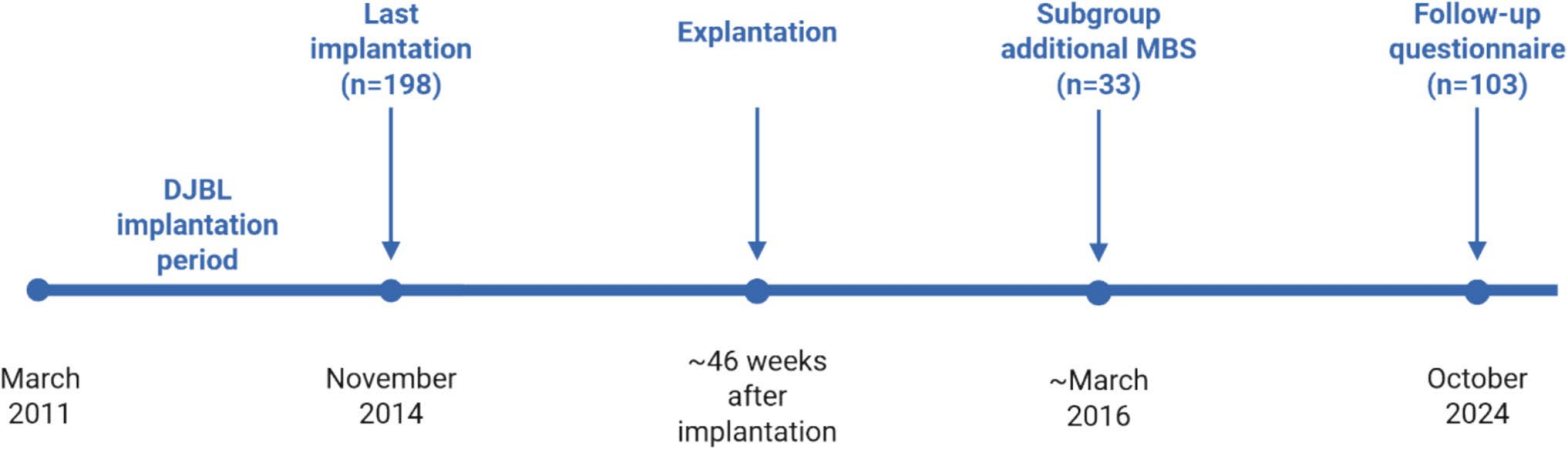
Overview of full time line from implantation to long-term follow DJBL: Duodenal-Jejunal Bypass Liner, MBS: Metabolic Bariatric Surgery

Mean DJBL implantation time was 46 (± 15) weeks; 128 patients (69.2%) completed the full 54-week period, while 57 patients (30.8%) had early removal (median 33 weeks) [1]. An overview of the full timeline from implantation to long-term follow-up is shown in Fig. 2. Approximately 10 years after explantation, weight improved from 107 ± 18 kg at baseline to 89.9 ± 16.9 kg (p = 0.0015). BMI and HbA1c also improved: BMI at explantation was 30.9 ± 4.3 kg/m^2^, and after 10 years 29.6 ± 4.0 kg/m^2^ (p = 0.004), and for HbA1c this was 61 mmol/mol and 60 ± 12.8 mmol/mol respectively (p = 0.043). (Table 2).

**Table 2:** Results survey group

	Survey group (n = 103)	*p* value
Complications T2D		
None	55 (53.9%)	
Retinopathy	7 (6.9%)	
Nephropathy	7 (6.9%)	
Neuropathy	14 (13.7%)	
Missing	20 (19.6%)	
Experience with DJBL		
Positive	48 (47.1%)	
Neutral	20 (19.6%)	
Negative	29 (28.4%)	
Missing	5 (4.9%)	
Underwent MBS	33 (32.0%)	
RYGB		
Gastric sleeve		
OAGB	29 (88%) 3 (9%) 1 (3%)	
Quality of life VAS EQ-5D-5L (0–100)	69.2 ± 19.2	
Lifestyle interventions		
Diet	12 (7.3%)	
Dietician	32 (19.5%)	
Combined lifestyle intervention	15 (9.1%)	
Exercise		
Times per week since DJBL	5.67 ± 4.28	*p* < 0.001
Times per week before DJBL	4.50 ± 4.193	
Minutes per movement since DJBL	54.27 ± 52.13	*p* = 0.015
Minutes per movement after DJBL	40.39 ± 31.18	

Values are means with standard deviation or n with percents in parentheses

*T2D* Type 2 Diabetes, *DJBL* Duodenal-Jejunal Bypass Liner, *MBS* Metabolic Bariatric Sugery, *OAGB* One Anastomosis Gastric Bypass, *VAS EQ-5D-5L* Visual Analogue Scale EuroQol-5Dimensions-5Levels.

### DJBL Experience

Of respondents, 48 patients (49.5%) had a positive experience with the DJBL, 29 patients (28.4%) negative, and 20 patients (19.6%) neutral (Table 2). Among those who gave an explanation for their experience (n = 92), 51 (55.4%) attributed it to the positive effects of the DJBL on weight or T2D, mainly those with positive experiences (84.3%).

Additionally, 13 (14.1%) patients reported no effect of the DJBL, often linked to neutral experiences (76.9%). Of those with negative experiences (29 patients, 31.5%), most of them (82.8%) attributed this to side effects or serious adverse events (SAEs), including pain and nausea (23.3%) and SAEs like liver abscess, pancreatitis and perforation (12.2%).

### Lifestyle Interventions

Following explantation of the DJBL, patients underwent various lifestyle interventions (Table 2). 32 patients (31%) received dietary counseling from a dietitian,12 patients (7.3%) are currently still following one or more dietary regimens, mostly low-carb (10 patients). Additionally, 15 patients (15%) participated in a combined lifestyle intervention program, which also included a focus on dietary habits.

Physical activity increased from 4.5 to 5.7 sessions/week (*p* = 0.033), with duration rising from 40 to 54 min (*p* = 0.025). Physical activity was defined broadly, including activities such as sports, walking a dog, and performing physical labor.

### Quality of Life

The mean quality of life score on the EQ VAS for the entire cohort was 69.2 ± 19.2. Most patients scored lower in severity (some or moderate problems) for the dimensions of mobility, usual activities and pain or discomfort. The scores for selfcare and anxiety/depression were generally more favorable.

### MBS versus non-MBS

Of all patients﻿, 32% (*n* = 33) underwent MBS following DJBL explantation, mostly RYGB (*n* = 29), but also sleeve gastrectomy (*n* = 3) and One Anastomosis Gastric Bypass (OAGB) (*n* = 1) (Table 3).

**Table 3:** Results 10 years after explantation in non-metabolic bariatric surgery versus metabolic bariatric surgery group

	Non MBS (*n* = 70)	MBS (*n* = 33)	p value
Age	63 ± 29	62 ± 5	p = 0.095
Experience DJBL			p = 0.243
Positive	36 (51%)	13 (39%)	
Neutral	12 (17%)	8 (24%)	
Negative	15 (21%)	12 (36%)	
Missing	7 (10%)	0 (0%)	
Body weight (kg)	92.6 ± 14.8	83.8 ± 15.1	p = 0.01
BMI (kg/m^2^)	30.2 ± 3.7	28.5 ± 3.6	p = 0.038
HbA1c	62.7 ± 11.2	53.7 ± 15.7	p = 0.022
Complications T2D			
None	32 (45,7%)	22 (66.7%)	p = 0.136
Retinopathy	6 (8.6%)	1 (3%)	p = 0.417
Nephropathy	5 (7.1%)	2 (6.1%)	p = 1.000
Neuropathy	12 (17.1%)	2 (6.1%)	p = 0.129
Quality of life VAS EQ-5D-5L score	66.1 ± 19.9	75.1 ± 16.5	p = 0.047

Values are means with standard deviation or n with percents in parentheses

*MBS* Metabolic Bariatric Sugery, *DJBL* Duodenal-Jejunal Bypass Liner, *T2D* Type 2 Diabetes, *VAS EQ-5D-5L* Visual Analogue Scale EuroQol-5Dimensions-5Levels

Ten years post-explantation, MBS patients had lower weight (83.9 vs. 92.6 kg, p = 0.01) and BMI (28.5 vs. 30.2 kg/m^2^, p = 0.038), compared to non-MBS patients (Table 3). Compared to their own BMI at the time of explantation (30.9 kg/m^2^), the MBS group showed a significant long-term reduction (p = 0.001), while the non-MBS group did not (30.2 vs. 30.9 kg/m^2^, p = 0.0177).

**Fig. 3:**
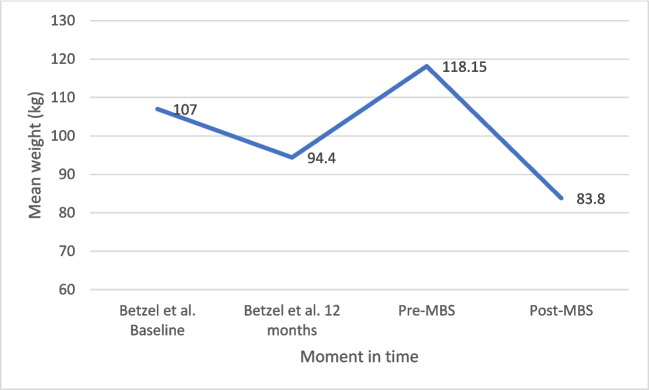
Mean body weight (kg) over time in patients who underwent metabolic bariatric surgery (MBS)

**Fig. 4:**
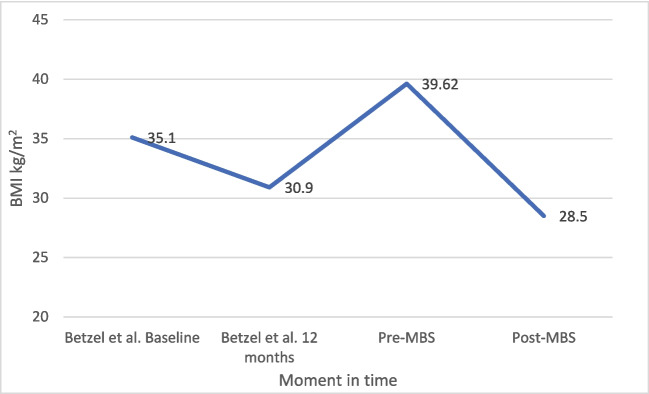
Mean BMI over time in patients with metabolic bariatric surgery (MBS)

The changes in weight and BMI in patients who underwent MBS are shown in Figs. 3 and 4. In the original study, a decrease in both weight and BMI was seen in the period the DJBL was in place (12 months) (107 to 94.4 kg, and 35.1 kg/m^2^ to 30.9 kg/m^2^, respectively). Following explantation until MBS, weight and BMI subsequently increased to 118.15 kg and 39.61 kg/m^2^, respectively. Following MBS, these values decreased again to 83.8 kg and 28.5 kg/m^2^, respectively.

HbA1c was higher in the non-MBS group (62.7 ± 11.2 mmol/mol) than in the MBS group (53.7 ± 15.7 mmol/mol) (p = 0.022). No difference was observed in the frequency of diabetes-related complications between the two groups.

Quality of life in patients who underwent MBS was higher than in those who did not (75.1 ± 16.5 vs. 66.1 ± 19.9).

When evaluating patients' experiences with the DJBL, no significant differences were observed between those who underwent MBS and those who did not. In the MBS group, 13 patients (39%) reported a positive experience with the DJBL, 8 patients (24%) a neutral experience, and 12 patients (36%) a negative experience. In the non-MBS group, these proportions were 36 patients (51%) positive, 12 patients (17%) neutral, and 15 patients (21%) negative (*p* = 0.243).

### GLP-1 agonist versus no GLP-1 agonist

At follow-up, 35 of 103 patients (34%) used GLP-1 agonists, vs. 26 of 198 (13%) during the original study (Table 4). GLP-1 users had a higher BMI (31.0 vs. 29.0 kg/m^2^, *p* = 0.022), but similar absolute weight (91.4 vs. 89.2 kg, *p* = 0.550). No significant differences were found in QoL or DJBL experience. Ten years post-explantation, BMI had significantly decreased in non-GLP-1 users compared to the end of the original study (*p* < 0.001), while no such change was observed in GLP-1 users (*p* = 0.944).

**Table 4:** Results 10 years after explantation no GLP-1 agonist versus GLP-1 agonist

	No GLP-1 agonist (*n* = 68)	GLP-1 agonist (*n* = 35)	*p* value
Age	62 ± 7	62 ± 14	p = 0.384
Experience DJBL			p = 0.172
Positive	28 (41.2%)	21 (60%)	
Neutral	16 (23.5%)	4 (11.4%)	
Negative	20 (29.4%)	9 (25.7%)	
Missing	4 (5.9%)	1 (2.9%)	
Body weight (kg)	89.2 ± 16.1	91.4 ± 18.3	p = 0.550
BMI (kg/m^2^)	29.0 ± 3.6	31.0 ± 4.6	p = 0.022
HbA1c	57.7 ± 15.1	62.0 ± 9.3	p = 0.235
Complications T2D			
None	35 (51.5%)	20 (57.1%)	p = 0.678
Retinopathy	6 (8.8%)	1 (2.9%)	p = 0.418
Nephropathy	6 (8.8%)	1 (2.9%)	p = 0.661
Neuropathy	7 (10.3%)	7 (2.0%)	p = 0.226
Quality of life VAS EQ-5D-5L score	72.3 ± 18.7	63.9 ± 19.1	p = 0.057

Values are means with standard deviation or n with percents in parentheses

GLP-1 agonist: Glucagon Like Peptide-1 agonist, *DJBL* Duodenal-Jejunal Bypass Liner, T2D: Type 2 Diabetes, VAS EQ-5D-5L: Visual Analogue Scale EuroQol-5Dimensions-5Levels

No significant differences were observed between the GLP-1 and non-GLP-1 groups regarding diabetes-related outcomes or retrospective experiences with the DJBL. Similarly, no significant differences were found in quality-of-life scores.

## Discussion

This is the first long-term follow-up study of patients who had a DJBL explanted approximately 10 years ago. We found that BMI 10 years after explantation was significantly higher than directly post-explantation (94.3 vs. 89.9 kg, p = 0.015). Additionally, 33 patients (32%) underwent MBS in the period following DJBL explantation.

Weight outcomes varied between subgroups. Patients who underwent MBS showed a significant BMI reduction afterwards (p = 0.001), while no significant difference was observed in the non-MBS group (p = 0.0177). Interestingly, BMI in the MBS group initially increased between DJBL explantation and MBS, even exceeding baseline BMI, before decreasing afterwards. This indicates that DJBL alone does not provide long-term weight reduction, whereas MBS may offer lasting effects, particularly if performed soon after DJBL removal.

HbA1c levels were lower at 10 years after DJBL explantation compared to directly after explantation, especially in the MBS group (p = 0.022). This aligns with evidence supporting MBS’s positive effect on metabolic control, particularly for patients with T2D [8]. Diabetes-related complications were rare and comparable between groups, suggesting a potential long-term benefit of DJBL, although diabetes complications may have predated MBS in some cases, limiting surgery’s impact on them.

GLP-1 agonist use increased from 13% during the original study to 34% at follow-up. Users had a higher BMI, consistent with guidelines recommending GLP-1 agonist use in patients with obesity. While no significant differences in weight, HbA1c, or diabetes complications were observed between users and non-users, GLP-1 agonists remain an important treatment for patients with elevated BMI and uncontrolled T2D [9].

Quality of life (QoL) scores were considerably lower in this study population compared to average for a healthy Dutch population, with a mean score of 81 [10]. Residual excess weight, T2D and other associated medical problems are likely to have contributed to these lower scores. In addition, in the original cohort study, 17% had adverse events, of which 3.7% were SAE’s [1]. In particular these SAE’s may have had an impact on the subsequent quality of life, as post-operative adverse events are known to affect patients’ well-being even in the (very) long term [11].

Subgroup analysis revealed higher QoL scores in the MBS group and the non-GLP-1 group compared to the non-MBS group and the GLP-1 group. Notably, these groups also had lower BMI, which may explain the observed differences in QoL. This is consistent with existing literature, showing that higher BMI is associated with lower QoL scores [12]. Additionally, medical problems associated with overweight or obesity could have contributed to the observed differences [13]. Another explanation could be the side effects commonly reported with GLP-1 agonists, such as gastrointestinal symptoms, headache, and injection side reactions, which may negatively impact QoL [14]. Interestingly, despite the potential for complications, the MBS group reported higher QoL scores, suggesting that the benefits of MBS may outweigh its potential challenges due to complications for many patients.

A previous study examined long-term outcomes of DJBL placement in 15 patients, over a 4-year follow-up period. In that study, 33% (5 of 15) of patients underwent MBS, and no significant differences in weight, BMI, or T2D parameters were observed compared to baseline [5]. However, those who underwent MBS were only followed until the procedure, with a median follow-up of 14 months. Given that this group represents 33% of all patients, this could introduce bias, as outcomes for these patients over a full 4-year period (without MBS) remain uncertain.

## Strengths and Limitations

A major strength of this study is its unique long-term follow-up duration of approximately 10 years after DJBL explantation, offering valuable insights into the durability of its effects and the subsequent treatment pathways. Additionally, the study incorporates multiple dimensions of health outcomes, including weight, metabolic control, lifestyle, and quality of life.

However, several limitations must be acknowledged. First, the results of this study were based on a questionnaire completed approximately 10 years after DJBL explantation, which may introduce recall bias. The long interval may have affected patients’ ability to accurately remember past events and outcomes [15]. Additionally, data on weight, quality of life and lifestyle factors, were self-reported. While self-reporting is common in long-term follow-up studies, it may introduce measurement bias and affect the accuracy of findings due to lack of objective verification.

Second, the response rate was 51%, raising concerns about non-response bias, which may affect the validity and generalizability of our findings. Despite efforts to reach non-respondents, there may be differences between those who responded and those who did not [16]. It is conceivable that patients who had a positive experience and had more favorable outcomes were more likely to respond than those with a negative experience, which could lead to an overrepresentation of beneficial effects [17]. Conversely, less favorable experiences may be underreported, which is a common challenge in observational studies relying on voluntary follow-up. This differential participation can introduce selection bias, thereby limiting the external validity of the study. Unfortunately, baseline data for non-responders were not available due to lack of consent for extended follow-up, preventing formal comparison.

Third, the absence of an external control group limits the ability to attribute observed outcomes exclusively to the DJBL, as confounding factors such as lifestyle changes, concurrent treatments, or natural disease progression may have contributed to the results.

Furthermore, information on the timing of GLP-1 initiation in relation to DJBL explantation or subsequent MBS was not available. As follow-up was based on a single cross-sectional, anonymized questionnaire, we were unable to retrieve detailed treatment trajectories or verify them against medical records. Consequently, it remains unclear whether GLP-1 use reflects treatment failure, an adjuvant strategy following MBS, or an independent management approach during follow-up. This limitation hampers the interpretation of GLP-1 outcomes within our cohort.

Despite its current unavailability in clinical practice due to earlier safety concerns, the DJBL may hold clinically relevance in the future as a bridging therapy before MBS. Surgery carries significant risks, especially in patients with severe obesity [18]. The DJBL, being a minimally invasive and reversible endoscopic procedure performed under conscious sedation, can help patients lose weight preoperatively and thereby reduce surgical risks [19]. It may also offer an option for those unwilling or unsuitable for surgery. While this study alone cannot define the DJBL’s definitive role, it contributes valuable insight into its possible utility within a multimodal treatment approach. Further research is needed to clarify its long-term benefits and position in bariatric care.

## Conclusion

This study highlights the transient effectiveness of DJBL as a weight loss intervention, with long-term outcomes largely dependent on subsequent treatment. MBS following DJBL explantation was associated with significant and sustained weight reduction, improved glycemic control, and better QoL, emphasizing the benefit of a multi-modal approach for durable outcomes. In contrast, patients who did not undergo further treatment experienced less weight loss and lower QoL. These findings suggest that combining DJBL with additional interventions, such as MBS, may enhance its long-term efficacy.

## Data Availability

No datasets were generated or analysed during the current study.
